# Does the effectiveness of neuraxial morphine administration vary across different patient populations, dosing strategies, and delivery techniques?

**DOI:** 10.3325/cmj.2025.66.235

**Published:** 2025-06

**Authors:** Nadija Gačo Rački, Miroslav Župčić, Sandra Graf Župčić, Valentino Rački, Tatjana Šimurina, Viktor Duzel

**Affiliations:** 1 Department of Anesthesiology, Intensive Care and Pain Medicine, Rijeka University Hospital Center, Rijeka, Croatia; 2 Department of Clinical Sciences II, Faculty of Health Studies, University of Rijeka, Rijeka, Croatia; *miroslav.zupcic@uniri.hr*; 3 Department of Neurology, University Hospital Center Rijeka, Rijeka, Croatia; 4 Faculty of Biotechnology and Drug Development, University of Rijeka, Rijeka, Croatia; 5 Department of Neurology, Faculty of Medicine, University of Rijeka, Rijeka, Croatia; 6 Department of Anesthesiology, Resuscitation and Intensive Care Medicine, Zadar General Hospital, Zadar, Croatia; 7 Department of Health Studies, University of Zadar, Zadar, Croatia; 8 Department of Anesthesiology, Resuscitation, Intensive Medicine and Pain Management, Faculty of Medicine, Josip Juraj Strossmayer University of Osijek, Osijek, Croatia; 9 Northern Hospital Epping, Melbourne, Victoria, Australia

The administration of neuraxial morphine for postoperative analgesia remains a cornerstone of modern anesthetic practice, as supported by its widespread use and proven efficacy in reducing postoperative pain and opioid requirements (1,2). Morphine, a hydrophilic opioid, produces prolonged analgesic effects when administered via neuraxial routes, including intrathecal and epidural administration (3). However, its clinical effectiveness varies considerably across different patient populations, dosing strategies, and delivery techniques. This variability raises the question if there is a discernible difference in the effectiveness of neuraxial morphine administration. A closer examination of the available literature provides insight into the factors contributing to these differences.

Neuraxial morphine provides longer analgesia primarily due to its hydrophilic nature, which allows slow diffusion in the cerebrospinal fluid and prolongs its effect at the opioid receptors in the dorsal horn of the spinal cord (2,4). Compared with lipophilic opioids, such as fentanyl or sufentanil, morphine produces slower-onset but more durable analgesia (2). While its clinical efficacy is not debated, the route of administration considerably influences its pharmacokinetics and associated adverse effects. Intrathecal morphine has been shown to achieve superior analgesia at lower doses compared with epidural administration. For instance, a single dose of intrathecal morphine not only provides effective pain relief but also reduces the intensity of postoperative pain and opioid consumption, facilitating a faster and higher-quality recovery following surgical interventions (1,3).

Laparoscopic procedures for colorectal cancers are associated with reduced postoperative pain compared with open surgical approaches. However, among neuraxial techniques, epidural administration of morphine generally provides more intense analgesia due to the possibility of continuous dosing. Despite this, a single intrathecal dose of morphine has demonstrated superior postoperative analgesia compared with intravenous opioid administration (2). Intrathecal morphine has the advantage of delivering prolonged analgesia without the need for repeated doses, as is common with intravenous or epidural opioids. This can be particularly advantageous in enhancing recovery by reducing opioid-related side effects, such as nausea, vomiting, and respiratory depression, which are often dose-dependent (2,5).

Dose-response relationships even more underline the difficulty of neuraxial morphine delivery. Although raising dosages improves analgesic efficacy, there is a ceiling effect beyond which adverse reactions show more clearly. The most frequent adverse reactions, which are dose-dependent, are pruritus, nausea, and vomiting. More important, though, is the possibility of delayed respiratory depression, especially in vulnerable patient groups or with greater doses of epidural morphine. These hazards call for cautious titration and patient monitoring, particularly in patients with compromised respiratory function or opioid sensitivity (5).

Factors that further influence the efficacy of neuraxial morphine are patient-specific characteristics. Morphine pharmacokinetics and pharmacodynamics depend on age, comorbidities, and opioid tolerance. Patients with chronic kidney disease, for instance, may have longer effects because of lowered morphine clearance; those who are opioid-tolerant may need more doses to reach appropriate analgesia. These differences highlight the need for customized dose policies tailored to every patient's clinical profile (4,5).

Compared with systemic opioid treatment, meta-analyses and systematic reviews repeatedly show that neuraxial morphine lowers postoperative opioid consumption and increases pain scores. The possibility for side effects emphasizes the need for a balanced approach even if its advantages are obvious. Appropriate dosages, close monitoring of high-risk patients, and a combination of neuraxial morphine with multimodal analgesic approaches to lower the total opioid exposure help to maximize the risk-benefit ratio (1). An observational study in fifty patients undergoing a pancreatectomy showed better results when epidural anesthesia with intrathecal morphine was combined with transversus abdominis plane (TAP) blocks compared with TAP blocks alone, with equivalent efficacy (6). Similarly, in 303 patients undergoing pancreatectomy, intrathecal morphine was superior to TAP blocks with reduced pain scores and opioid requirements (7). Finally, in a retrospective study of 427 patients who underwent upper gastrointestinal surgery, thoracic epidural analgesia was superior to intrathecal morphine, which was superior to peripheral blocks, in terms of the required postoperative opioid use and pain control (Table 1) (8).

**Table 1:** Studies comparing intrathecal morphine administration with other analgesic methods*

Study author	Number of patients	Morphine dose	Type of surgical procedure	Main conclusion
Koning et al (1)	56	300 μg with bupivacaine	Laparoscopic segmental colonic resections	Intrathecal morphine significantly reduced postoperative pain and opioid use within ERAS programs, enhancing recovery speed.
El-Mallah (6)	50	Intrathecal morphine (single dose, no specifics)	Open pancreatoduodenectomy	Intrathecal morphine is comparable to epidural analgesia in efficacy and superior to TAP blocks alone, with shorter Foley removal time.
Pirie et al (8)	427	Median dose of 350 μg	Upper gastrointestinal surgery (open/laparoscopic)	Intrathecal morphine reduced opioid consumption compared with non-neuraxial analgesia, although effects were less sustained than those of epidural analgesia
Lokin et al	40	60 μg/mL in combination with bupivacaine	Laparoscopic colorectal surgery	Intrathecal morphine provided effective analgesia for 24 hours post-surgery but required adjunct non-opioid analgesics to reduce pain.
Boisen et al (7)	303	100-200 μg	Pancreatic surgery	Intrathecal morphine was associated with reduced pain scores and opioid requirements compared with peripheral nerve blocks in pancreatic surgery

*Abbreviations: ERAS – enhanced recovery after surgery, TAP – transverse abdominis plane.

In conclusion, while neuraxial morphine is effective for postoperative analgesia, its clinical outcomes vary depending on the route of administration, dosing strategies, and patient-specific factors. Intrathecal morphine appears to provide more effective analgesia at lower doses compared with epidural administration, but both routes require careful dose titration to minimize adverse effects. Furthermore, single-dose intrathecal morphine has demonstrated advantages in reducing postoperative pain intensity and opioid requirements, facilitating faster recovery, especially in minimally invasive procedures such as laparoscopic colorectal surgeries. Further prospective randomized trials are needed to establish standardized protocols that optimize its analgesic efficacy while mitigating the risks. Addressing these questions will enable clinicians to adapt neuraxial morphine administration to maximize its benefits and improve patient outcomes.
